# Reply to "comment on: Socioeconomic inequities in prostate cancer care: private *versus* public treatment settings pose a significant impact on overall survival"

**DOI:** 10.31744/einstein_journal/2026CE2477

**Published:** 2026-04-24

**Authors:** Amanda Caroline de Souza Costa, Fernando Korkes, Jose Augusto Rinck, Frederico Timóteo Silva Cunha, Luciana Holtz de Camargo Barros, Stênio de Cássio Zequi, Maria Nirvana da Cruz Formiga

**Affiliations:** 1 AC Camargo Cancer Center São Paulo SP Brazil AC Camargo Cancer Center, São Paulo, SP, Brazil.; 2 Centro Universitário FMABC Santo André SP Brazil Centro Universitário FMABC, Santo André, SP, Brazil.; 3 Hospital Israelita Albert Einstein São Paulo SP Brazil Hospital Israelita Albert Einstein, São Paulo, SP, Brazil.; 4 Instituto Oncoguia São Paulo SP Brazil Instituto Oncoguia, São Paulo, SP, Brazil.

Dear Editor,

We would like to thank D’Elia et al. for their thoughtful and constructive comments regarding our article, "Socioeconomic inequities in prostate cancer care: private versus public treatment settings pose a significant impact on overall survival" published in einstein (São Paulo).^(1)^ We fully agree with the authors regarding the relevance and urgency of this discussion.

With this study, our primary objective was to document, using real-world data from a single tertiary center, a situation that unfortunately reflects a broader and persistent reality across different health systems in Brazil. By minimizing institutional variability and focusing on differences largely driven by payer status, we sought to highlight how structural inequities in access to diagnostic tools and life-prolonging therapies may translate into meaningful survival gaps.

We agree that moving from description to intervention is essential. Our intention in publishing these data was precisely to stimulate a more mature and pragmatic discussion regarding feasible strategies to mitigate disparities within resource-constrained settings. This includes critical reflection on treatment-line sequencing and cost-conscious approaches.

Examples of such strategies may include (a) the use of orchiectomy as a definitive androgen-deprivation modality to reduce long-term costs and logistical barriers;^(2)^ (b) the earlier introduction of abiraterone with dose optimization strategies aimed at improving pharmacologic absorption and cost-effectiveness;^(3)^ (c) the selective use of triplet therapy including docetaxel in clearly defined high-volume or synchronous multimetastatic disease scenarios, where evidence suggests greater absolute benefit. (d) Initiatives to stimulate patient advocacy and public policy interventions.^(4)^ In this regard, *Instituto Oncoguia* has been an important partner in addressing this issue.

These discussions are not intended to lower standards of care, but rather to explore context-adapted strategies that may expand access to effective therapies while preserving clinical benefit. In parallel, optimizing resource allocation could create greater budgetary flexibility to enable incorporation of additional novel agents into public formularies.

We acknowledge the limitations highlighted by the authors, including residual confounding and the need for more granular analyses of drug exposure, sequencing, and time-on-therapy. We agree that future multicenter and registry-based studies, as well as methodological approaches incorporating time-dependent analyses, will be essential to better disentangle the mediators of survival differences and identify actionable system-level levers.

Ultimately, we believe that transparent documentation of inequities is a necessary first step toward policy change. We hope our findings contribute to ongoing national discussions regarding drug incorporation timelines, access to clinical trial, and structural reforms within the Brazilian Unified Health System (SUS - *Sistema Único de Saúde*).

We thank the authors again for their engagement and for advancing this important debate.

## Data Availability

The content is already available.
